# Booster mRNA Vaccination Prevents Breakthrough Severe COVID‐19 Infections

**DOI:** 10.1002/jmv.71026

**Published:** 2026-06-18

**Authors:** Harika Öykü Dinç, Hayriye Kirkoyun. Uysal, Günay Can, Beyhan Budak, Ferhat Osman Daşdemir, Elif Keskin, Okan Aydoğan, İlker İnanç Balkan, Rıdvan Karaali, Suat Saribaş, Sevgi Ergin, Neşe Saltoğlu, Bekir Kocazeybek

**Affiliations:** ^1^ Department of Medical Microbiology, Faculty of Medicine Üsküdar University Istanbul Turkey; ^2^ Department of Medical Microbiology, Faculty of Medicine Istanbul University Istanbul Turkey; ^3^ Department of Public Health, Cerrahpaşa Medical Faculty Istanbul University‐Cerrahpaşa Istanbul Turkey; ^4^ Department of Infectious Diseases and Clinical Microbiology, Cerrahpaşa Medical Faculty Istanbul University‐Cerrahpaşa Istanbul Turkey; ^5^ Department of Medical Microbiology, Cerrahpaşa Medical Faculty Istanbul University‐Cerrahpaşa Istanbul Turkey; ^6^ Department of Medical Microbiology, Faculty of Medicine Istanbul Medipol University Istanbul Turkey

**Keywords:** antibody, BNT162b2, COVID‐19, ELISA, SARS‐CoV‐2

## Abstract

This study aimed to assess the humoral immune response following administration of a BNT162b2 mRNA COVID‐19 booster dose and to evaluate its association with the development of SARS‐CoV‐2 infection after vaccination. Between July 2021 and February 2022, serum samples were collected from 313 individuals 28 days after receiving a COVID‐19 booster dose. Quantitative SARS‐CoV‐2 IgG antibodies directed against the receptor‐binding domain (RBD) of the spike protein were measured using a chemiluminescent microparticle immunoassay. Neutralizing antibody activity, defined as inhibition of the RBD–ACE2 interaction, was evaluated semi‐quantitatively by a competitive ELISA. Participants were followed for 6 months after the final dose to identify confirmed SARS‐CoV‐2 infections. The cohort included 97 males (31%) and 216 females (69%), with a mean age of 39.43 years. The median SARS‐CoV‐2 IgG antibody level at day 28 was 7713.3 AU/mL, and the median neutralizing antibody inhibition rate was 99.2%. Antibody levels were not significantly associated with age or comorbidities. Booster vaccination induced a strong humoral immune response in all participants. However, the occurrence of post‐vaccination infections despite high antibody levels suggests immune escape by emerging variants. While vaccines may not fully prevent infection, they provide substantial protection against severe disease and mortality.

## 1 Introduction

Over the past 3 years, various types of vaccines, including mRNA, inactivated, protein subunit, and adenovirus vector vaccines have been developed and continue to be widely used worldwide with the aim of effectively preventing COVID‐19, reducing mortality, and achieving herd immunity [1, 2, 3, 4]. However, the presence of different mutations in SARS‐CoV‐2 leading to new variants circulating since its emergence in 2019 has posed challenges in the control of COVID‐19. The ongoing emergence of new variants has sustained the risk of infection. Despite the high levels of humoral immune response conferred by the current vaccines and maintained through booster doses, COVID‐19 cases continue to be observed. During the early phase of booster dose implementation, the Omicron variant was the predominant circulating strain and was characterized by extensive mutations in the spike protein, including more than 15 alterations within the receptor‐binding domain (RBD), the principal target of neutralizing antibodies [5]. These structural changes have been shown to reduce the susceptibility of SARS‐CoV‐2 variants to both monoclonal and polyclonal antibody‐mediated neutralization [6, 7]. This decrease in antibody efficacy has resulted in numerous new infections and cases of reinfection, contributing to the spread of the virus in both non‐immune and immune individuals [8].

Previous studies have consistently reported a markedly reduced neutralizing antibody response against the Omicron variant in vaccinated individuals compared with responses observed against the wild‐type SARS‐CoV‐2 strain and the Delta (B.1.617.2) variant. While the variants of interest (VOIs) identified as EG.5 and its subtypes in the latest risk assessment by the WHO have not shown any changes in disease severity, they have demonstrated the ability to escape immune responses (‐). However, it has been reported that neutralizing antibody activity could be effective with the administration of booster doses [9, 10, 11]. In a prospective cohort study conducted in Germany based on the follow‐up of individuals who received mRNA vaccines before and after booster doses, it was found that study participants with anti‐spike antibody levels of 2816.0 BAU/mL or lower had a twofold higher risk of infection compared to individuals who exceeded this threshold (OR: 2.12, 95% CI: 1.24–3.58) [12].

In this context, our study aimed to retrospectively analyze the data arising from the ongoing COVID‐19 vaccination process at our center, with the objective of conducting an analysis to determine the risk of infection based on the neutralizing antibody response after the administration of booster doses of the COVID‐19 vaccine during the period when the Omicron variant was predominant.

## 2 Materials and Methods

### 2.1 Study Design and Participants

This study was designed as a retrospective, single‐center observational cohort analysis conducted at the Cerrahpaşa Faculty of Medicine COVID‐19 Vaccination Clinic. A total of 313 volunteers who received a COVID‐19 booster dose between July 2021 and February 2022 were included. Blood samples were obtained 28 days after the booster dose from participants who had voluntarily consented to the use of their samples for research purposes. Demographic characteristics, including age, sex, clinical symptoms, and comorbid conditions, were recorded using standardized follow‐up forms. In addition, participants were monitored for 6 months after the final vaccination dose to identify confirmed SARS‐CoV‐2 infections through face‐to‐face interviews and polymerase chain reaction (PCR) record review. The overall study approach and immunological assessment strategy were consistent with previously published cohort studies conducted at the same center [13].

### 2.2 Serological Tests

#### 2.2.1 Quantitative SARS‐CoV‐2 IgG Measurement

Quantitative detection of SARS‐CoV‐2‐specific IgG antibodies directed against the RBD of the spike protein S1 subunit was performed using a chemiluminescent microparticle immunoassay (CMIA) (ARCHITECT IgG II Quant, Abbott Laboratories, USA). Analyzes were carried out on Architect and Alinity platforms in accordance with the manufacturer's instructions. Antibody concentrations were initially expressed in arbitrary units per milliliter (AU/mL) and subsequently converted to binding antibody units per milliliter (BAU/mL) using the World Health Organization (WHO) conversion factor (0.142). Antibody concentrations ≥ 50 AU/mL (≥ 7.1 BAU/mL) were considered seropositive [14]. Accordingly, concentrations of 50 AU/mL or 7.1 BAU/mL and above were considered positive. Previous validation studies have demonstrated a high level of agreement between this assay and the plaque reduction neutralization test (PRNT), supporting its reliability in evaluating post‐booster antibody responses [13, 15]. In this context, an antibody concentration of 1050 AU/mL has been reported to correspond to a PRNT dilution of 1:80 [15].

### 2.3 Surrogate Neutralization Test

Neutralizing antibody activity was assessed using a surrogate virus neutralization test based on a competitive enzyme‐linked immunosorbent assay (ELISA) (SARS‐CoV‐2 NeutraLISA, Euroimmun, Lübeck, Germany). This assay measures the ability of serum antibodies to inhibit the interaction between the SARS‐CoV‐2 S1‐RBD and the human ACE2 receptor. Analyzes were performed using the EUROIMMUN Analyzer. Neutralization capacity was calculated as the percentage of inhibition (IH%), with values < 20% considered negative, 20%–35% considered borderline, and ≥ 35% considered positive, in accordance with the manufacturer's recommendations. The surrogate neutralization assay demonstrates high compatibility with PRNT and has been validated in previous cohort studies evaluating booster‐induced immunity [13]. Validation studies have shown that the EUROIMMUN SARS‐CoV‐2 NeutraLISA assay demonstrates a high level of agreement with the PRNT, with a reported compatibility rate of 98.6%, supporting its use as a reliable surrogate neutralization method [15].

### 2.4 Statistical Analysis

Statistical analyzes were conducted using IBM SPSS Statistics version 21. Categorical variables were expressed as frequencies and percentages, while continuous variables were reported as medians with interquartile ranges (IQR, 25th–75th percentiles). Group comparisons were performed using the chi‐square test or Fisher's exact test for categorical variables, and the Student's *t*‐test, Mann–Whitney *U* test, or Kruskal–Wallis test for continuous variables, as appropriate. Correlations between antibody levels and clinical parameters were evaluated using Spearman's correlation analysis. A two‐sided *p*‐value < 0.05 was considered statistically significant.

## 3 Results

Among the 313 participants who received the BNT162b2 mRNA COVID‐19 vaccine as the final dose, vaccination schedules were distributed as follows: 27 participants received 2 doses of BNT162b2 followed by 1 additional BNT162b2 dose, 192 received 2 doses of CoronaVac followed by 1 dose of BNT162b2, 81 received 2 doses of CoronaVac followed by 2 doses of BNT162b2, and 13 received 2 doses of CoronaVac followed by 3 doses of BNT162b2. The study population consisted predominantly of female participants (*n* = 216, 69%), while the remaining participants were male (*n = *97, 31%). No statistically significant differences were observed in terms of gender distribution (Table 1).

**Table 1:** Assessment of demographic data by vaccine groups in the study population.

	3 dose Biontech	2 dose CoronaVac + 1 Biontech	2 dose CoronaVac + 2 Biontech	2 dose CoronaVac + 3 Biontech	Total	*p*
Demographic data	*n*: 27 (%)	*n*: 192 (%)	*n*: 81 (%)	*n*: 13 (%)	*n*: 313 (%)	
**Gender**						
Male	7 (25.9)	65 (33.9)	21 (25.9)	4 (30.8)	97 (31)	0.566
Female	20 (74.1)	127 (66.1)	60 (74.1)	9 (69.2)	216 (69)	
**Age groups**						
< 30	7 (25.9)	42 (21.9)	16 (19.8)	3 (23.1)	68 (21.7)	0.482
30–39	10 (37)	44 (22.9)	23 (28.4)	6 (46.2)	83 (26.5)	
40–49	8(29.6)	34.9 (29)	29 (35.8)	2 (15.4)	106 (33.9)	
50 years and older	2 (7.4)	39 (20.3)	13 (16)	2 (15.4)	56 (17.9)	
**Post‐vaccination infection**						
No	18 (66.7)	116 (60.4)	56 (69.1)	13 (100)	203 (64.9)	0.012*
Yes	9 (33.3)	76 (39.6)	25 (30.9)	0 (0)	110 (35.1)	
**Comorbidity**						
No	22 (81.5)	140 (72.9)	60 (74.1)	9 (69.2)	231 (73.8)	0.790
Yes	5 (18.5)	52 (27.1)	21 (25.9)	4 (30.8)	82 (26.2)	
**Autoimmune disease**						
No	27 (100)	188 (97.9)	78 (96.3)	13 (100)	306 (97.8)	0.084
Yes	0 (0)	4 (2.1)	3 (3.7)	0 (0)	7 (2.2)	
**Malignity**						
No	27 (100)	190 (99)	81 (100)	13 (100)	311 (99.4)	—
Yes	0 (0)	2 (1)	0 (0)	0 (0)	2 (0.6)	
**Diabetes mellitus**						
No	27 (100)	186 (96.9)	76 (93.8)	13 (100)	302 (96.5)	—
Yes	0 (0)	6 (3.1)	5 (6.2)	0 (0)	11 (3.5)	
**Hypertension**						
No	26 (96.3)	183 (95.3)	75 (92.6)	13 (100)	297 (94.9)	—
Yes	1 (3.7)	9 (4.7)	6 (7.4)	0 (0)	16 (5.1)	
**Hypothyroidism**						
No	23 (85.2)	183 (95.3)	72 (88)	12 (92.3)	290 (92.7)	—
Yes	4 (14.8)	9 (4.7)	9 (11.1)	1 (7.7)	23 (7.3)	
**Neurological disease**						
No	27 (100)	192 (100)	79 (97.5)	13 (100)	311 (99.4)	—
Yes	0 (0)	0 (0)	2 (2.5)	0 (0)	2 (0.6)	
**Heart failure**						
No	27 (100)	188 (97.9)	80 (98.8)	12 (92.3)	307 (98.1)	—
Yes	0 (0)	4 (2.1)	1 (1.2)	1 (7.7)	6 (1.9)	
**Lung disease**						
No	26 (96.3)	192 (100)	79 (97.5)	13 (100)	310 (99)	—
Yes	1 (3.7)	0 (0)	2 (2.5)	0 (0)	3 (1)	
**Allergic diseases**						
No	27 (100)	180 (93.7)	71 (87.7)	12 (92.3)	290 (92.7)	—
Yes	0 (0)	12 (6.3)	10 (12.3)	1 (7.7)	23 (7.3)	

Within 6 months following the final vaccination dose, 203 participants (64.9%) did not experience laboratory‐confirmed COVID‐19 infection, indicating a significantly high level of protection against SARS‐CoV‐2 in the study cohort (*p* = 0.012; Table 1). When vaccine regimens were analyzed individually, the highest protection rates were observed in the 2 CoronaVac + 2 BNT162b2 and 2 CoronaVac + 3 BNT162b2 groups. Face‐to‐face clinical interviews revealed that all post‐vaccination breakthrough infections were limited to mild or moderate disease severity.

In participants vaccinated with 2 doses of BNT162b2 followed by 1 booster dose, the median SARS‐CoV‐2 IgG antibody concentration measured 28 days after the final dose was 7713.3 AU/mL, accompanied by a neutralizing antibody inhibition rate of 99.2%. In contrast, individuals who received heterologous vaccination schedules demonstrated median IgG antibody levels of 8162.4 AU/mL, 9567.7 AU/mL, and 9638.5 AU/mL, respectively. Corresponding neutralization inhibition percentages were 98.9%, 99.5%, and 99.4%. Comparative analysis across vaccination profiles showed no statistically significant differences in SARS‐CoV‐2 IgG concentrations or neutralizing antibody activity (Table 2; *p* > 0.05).

**Table 2:** Evaluation of SARS‐CoV‐2 IgG and neutralizing antibody responses in blood samples taken after the final dose according to different vaccination profiles.

Serological tests	3 dose Biontech	2 dose CoronaVac + 1 Biontech	2 dose CoronaVac + 2 Biontech	2 dose CoronaVac + 3 Biontech	*p*
	Median (IQR25–75)	Median (IQR25–75)	Median (IQR25–75)	Median (IQR25–75)	
SARS‐CoV‐2 IgG, AU/mL	7713.3 (3668.3–13849.5)	8162.4 (4778.7–14817.9)	9567.7 (3837.8–16197.1)	9638.5 (5038.8–17769.2)	0.586
nAb, IH%	99.2 (97.1–99.6)	98.9 (94.1–99.5)	99.5 (98.3–99.6)	99.4 (99.0–99.6)	0.343

**Figure 1:**
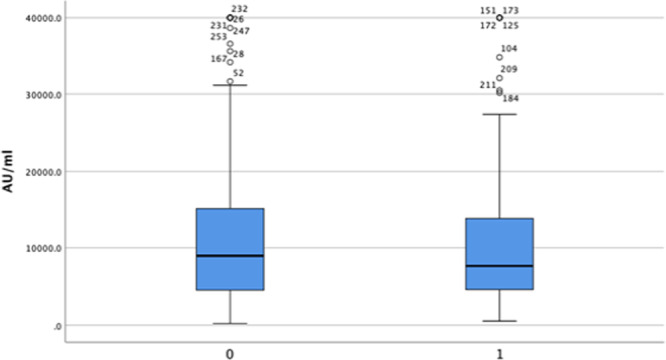
SARS‐CoV‐2 IgG response (AU/mL) according to post‐vaccination infection status. *Figure 1 illustrates SARS‐CoV‐2 IgG antibody concentrations according to previous SARS‐CoV‐2 infection history (0 = no previous infection; 1 = previous infection).

**Figure 2:**
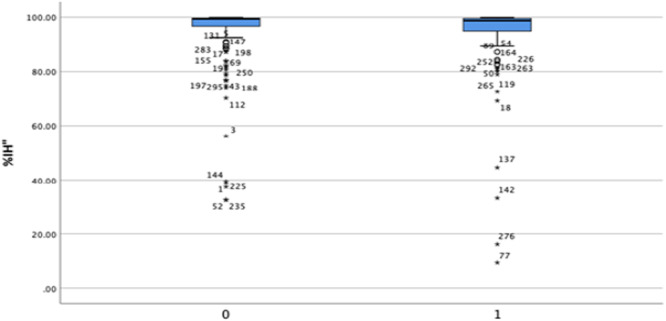
Comparison of neutralizing antibody inhibition (IH%) by post‐vaccination infection status. *Figure 2 illustrates neutralizing antibody inhibition percentages (IH%) according to SARS‐CoV‐2 infection history before the last vaccine dose (0 = no previous infection; 1 = previous infection).

When median SARS‐CoV‐2 IgG antibody levels were evaluated according to sex, male participants exhibited significantly higher antibody titers than females. No meaningful differences were observed when antibody responses were compared across age groups or in relation to comorbid conditions (Table 3; *p* > 0.05). Seventy‐one participants (22.7%) reported a history of COVID‐19 infection at least 3 months prior to receiving the final vaccine dose. These individuals displayed significantly lower median SARS‐CoV‐2 IgG antibody levels compared with participants without prior infection (Table 3; *p* = 0.04). Median antibody concentrations were 7622.4 AU/mL in those with post‐vaccination infection and 8951.5 AU/mL in those without (Figure 1). However, neutralizing antibody inhibition rates did not differ significantly between the two groups (*p* > 0.05) (Figure 2). Further analyzes demonstrated no significant associations between neutralizing antibody activity and sex, age, COVID‐19 history, or comorbidities (*p* > 0.05; Table 3).

**Table 3:** Assessment of SARS‐CoV‐2 IgG and neutralizing antibody responses in study group cases by demographic data.

Demographic data	SARS‐CoV‐2 IgG, AU/mL	*p*	nAb, IH%	*p*
	Median (IQR 25–75)		Median (IQR 25–75)	
**Gender**				
Male	11320.3 (5051.3–18369.6)	0.008	99.1 (95.8–99.6)	0.766
Female	7483.3 (4120.4–13696.9		99.3 (96.4–99.5)	
**Age groups**				
< 30	7476.8 (4814.1–13961.2)	0.815	99.0 (95.3–99.5)	0.437
30–39	8240.3 (4157.4–15276.7)		99.3 (96.0–99.6)	
40–49	8862.2 (4408.4–16243.6)		99.3 (96.9–99.5)	
50 years and older	10068.6 (4988.2–14354.9)		99.0 (95.0–99.6)	
**Post‐vaccination infection**				
No	8951.5 (4368.3–15245.4)	0.470	99.3 (96.6–99.6)	0.054
Yes	7622.2 (4522.4–13912.4)		98.7 (94.9–99.5)	
**Pre‐vaccination infection**				
No	9405.5 (4643.2–15939.1)	0.037	99.2 (95.7–99.5)	0.284
Yes	7021.3 (3590.2–13602.4)		99.4 (97.4–99.6)	
**Comorbidity**				
No	8342.5 (4524.4–15593.4)	0.748	99.3 (96.1–99.6)	0.770
Yes	8872.2 (4344.5–13849.4)		99.2 (95.8–99.5)	
**Autoimmune disease**				
No	8458.7 (4495.4–14980.7)	0.720	99.2 (96.1–99.5)	0.787
Yes	7560.9 (4695.0–10475.7)		99.2 (83.7–99.5)	
**Malignity**				
No	8470.2 (4516.6–14962.3)	0.610	99.2 (96.1–99.5)	0.987
Yes	6785.8 (5487.2)		98.9 (98.4)	
**Diabetes mellitus**				
No	8119.1 (4416.1–14494.8)	0.06	99.2 (96.0–99.5)	0.190
Yes	12831.9 (10475.7–17670.4)		99.4 (99.2–99.6)	
**Hypertension**				
No	8240.3 (4400.2–14604.6)	0.116	99.2 (95.9–99.5)	0.102
Yes	12007.0 (7591.5–22456.5)		99.5 (98.3–99.6)	
**Hypothyroidism**				
No	8687.9 (4524.7–15355.8)	0.238	96.1 (99.2–99.5)	0.909
Yes	7560.9 (3590.2–11577.8)		99.0 (95.6–99.6)	
**Neurological diseases**				
No	8447.3 (4516.6–14962.3)	0.931	99.2 (99.5–96.1)	0.597
Yes	9018.3 (7560.9)		89.2 (78.9)	
**Heart failure**				
No	8447.3 (4516.6–15036)	0.870	99.2 (96.3–99.5)	0.253
Yes	9653.3 (4462.2–12724.1)		95.3 (77.7–99.6)	
**Lung diseases**				
No	8458.7 (4522.4–14980.7)	0.564	99.2 (96.3–99.5)	0.347
Yes	7560.9 (4368.3)		78.9 (39.5)	
**Allergic diseases**				
No	8458.7 (4495.4–14980.7)	0.526	99.2 (95.9–99.5)	0.06
Yes	7874.3 (6650.9–14316.2)		99.5 (98.9–99.6)	

## 4 Discussion

In this study, where the humoral immune response parameters following the booster dose of the COVID‐19 vaccine were evaluated along with the monitoring of SARS‐CoV‐2 infection in the post‐vaccination period, it was observed that higher SARS‐CoV‐2 IgG titers were obtained as a result of heterologous vaccine administration, although there was no statistically significant difference. However, satisfactory levels of neutralizing antibody inhibition percentages were obtained in all vaccine profiles. During the follow‐up period, omicron and its subvariants were predominant in circulation. Over the course of follow‐up, 35.1% of participants experienced the infection with mild to moderate symptoms. When evaluating the data obtained from our study, it was concluded that post‐vaccination infection, SARS‐CoV‐2 IgG titers, and neutralizing antibody inhibition percentages were not determining criteria. However, neutralizing antibody responses observed after vaccination should be interpreted differently from those induced by natural infection. While natural infection involves viral replication and prolonged antigen exposure, vaccination stimulates immune responses in the absence of active infection and primarily aims to induce protective memory and effector immune cells. Therefore, differences in neutralization activity between previously infected and infection‐naïve vaccinated individuals may reflect distinct immunological mechanisms rather than differences in vaccine efficacy alone. Furthermore, the kinetics of humoral responses may present as monophasic or biphasic patterns depending on prior antigen exposure and individual immune history.

In a study conducted during the period when the delta variant was predominant in Austria, humoral and cellular immunological parameters potentially associated with protection against SARS‐CoV‐2 infection were monitored in 2760 participants for a 6‐month follow‐up period after receiving two doses of the BNT162b2 vaccine. Post‐vaccination, 2374 (86.0%) of the 2760 participants were found to be seropositive for neutralizing antibodies in surrogate neutralization tests. A strong positive correlation was demonstrated between surrogate neutralizing antibodies and Anti‐Spike IgG tests. During an average follow‐up period of 5.9 months, 68 (2.5%) participants were reported to have post‐vaccination infections. In conclusion, it was shown that high concentrations of binding and neutralizing antibodies after two doses of BNT162b2 vaccine were associated with a reduced risk of SARS‐CoV‐2 infection. However, no significant results were obtained in the assessment of cellular immune responses. Compared to our study, the percentage of post‐vaccination infections is quite low. This may be primarily attributed to the differences in the dominant variants during the periods in which both studies were conducted.

The emergence of SARS‐CoV‐2 variants has led to successive pandemic waves, posing significant public health challenges [16, 17, 18, 19, 20]. Studies have demonstrated that Omicron is more transmissible than other variants. The Omicron variant, characterized by 32 mutations in the spike protein region, is known to evade the immune response generated by wild‐type infection or vaccination [21]. However, studies have shown a significant decrease in neutralization activity against Omicron in serum samples from individuals who have had COVID‐19 and/or have been vaccinated [22, 23, 24, 25]. Therefore, to provide protective immunity against constantly evolving variants, the importance of booster doses in vaccination strategies has been emphasized.

A study reported from the UK highlighted the inadequacy of two doses of BNT162b2 or ChAdOx1 nCoV‐19 vaccines in providing protection against the Omicron variant. In the study, among individuals initially receiving BNT162b2, vaccine effectiveness was shown to be 67.2% (95% CI, 66.5–67.8) against infection within 2 to 4 weeks after the booster dose of BNT162b2 and then decreased to 45.7% (95% CI, 44.7–46.7) at 10 or more weeks after the booster. It was reported that the administration of booster doses with BNT162b2 or mRNA‐1273 resulted in a significant increase in protection against infection, but a decline over time was observed [26]. A review encompassing 52 studies that investigated post‐booster vaccine effectiveness revealed that the effectiveness of vaccines against symptomatic infection caused by the Omicron variant remained lower, even after booster doses, for all COVID‐19 vaccines compared to the Delta variant [27].

In parallel with these findings, recent studies have shown that bivalent and variant‐adapted vaccines, particularly those including XBB lineages, can induce broader neutralizing antibody responses and improved cross‐protection against emerging variants [28]. Chen et al. reported that the Tri‐XBB.1.5 vaccine demonstrated superior antibody durability compared to Bi‐Omi‐XBB and Tetra‐XBB.1 formulations, suggesting formulation‐dependent differences in long‐term immunogenicity [28]. In the same study, SARS‐CoV‐2 infection occurred in 27 participants during 6 months of follow‐up, including 11 (12.2%) confirmed and 16 (17.8%) probable cases, with relatively higher infection rates observed among recipients of bivalent and tetravalent vaccines [28]. These findings indicate that although updated vaccines enhance immune responses, they cannot fully prevent infection due to ongoing viral evolution and immune escape.

Another important consideration is the waning of antibody responses over time. Although high antibody levels were detected in our cohort, it has been demonstrated that SARS‐CoV‐2‐specific antibody responses decline within 3–6 months after vaccination, likely due to insufficient development of long‐lived plasma cells [29]. Similarly, studies evaluating XBB‐containing vaccines have reported a 2.5–4.6‐fold decrease in neutralizing antibody levels within 6 months, despite persistence of detectable immunity [29]. These findings suggest that antibody levels alone may not fully reflect long‐term protection and that cellular immunity also plays a critical role.

Previous studies have demonstrated that repeated antigen exposure through SARS‐CoV‐2 infection and vaccination can substantially enhance humoral immunity, although the magnitude and persistence of this response vary considerably among individuals. In a population‐based study, vaccinated convalescent individuals exhibited significantly higher antibody concentrations than individuals with infection or vaccination alone, suggesting an additive effect of cumulative immunological stimulation [30]. Longitudinal follow‐up data further showed that while some individuals maintained high antibody levels for prolonged periods, others experienced a marked decline despite booster vaccinations. These findings highlight the heterogeneity of humoral immune responses and suggest that fixed revaccination schedules may not adequately reflect individual immune status. Monitoring antibody kinetics may therefore contribute to more personalized booster vaccination strategies [31].

In addition, hybrid immunity resulting from the combination of natural infection and vaccination has been shown to provide stronger and more durable immune responses compared to vaccination alone. Guang et al. demonstrated that antibody levels following natural infection were higher than those induced by vaccination and could persist for more than 1 year [32]. This may partially explain variations in immune responses observed in individuals with prior infection in our study.

In our study, we could not establish a clear association between humoral immune parameters and post‐vaccination infection. However, despite high antibody levels, the occurrence of infection supports the role of immune escape mechanisms, particularly associated with Omicron and its subvariants. Nevertheless, the mild disease course observed in infected individuals suggests that booster doses provide strong protection against severe disease and mortality.

Although our findings are largely consistent with the literature, our study provides valuable real‐world data from a period when monovalent vaccines were predominantly used. Considering the continuous evolution of SARS‐CoV‐2, these data are important for understanding immune responses to earlier vaccination strategies and may contribute to preparedness for future pandemics and coronavirus outbreaks.

The most important limitation of our study is that it reflects a period during which monovalent vaccines were predominantly administered. Therefore, the effects of bivalent and variant‐specific vaccines could not be evaluated. Given that newer vaccines provide broader neutralization against emerging variants, our findings may not be fully generalizable to current vaccination strategies. Additionally, although humoral immune responses were evaluated with highly compatible serological tests (98%–100% agreement with PRNT), cellular immune responses could not be assessed. Another limitation is the absence of baseline antibody measurements before booster vaccination. Since antibody levels were assessed only 28 days after the booster dose, pre‐existing humoral immunity could not be determined. In addition, asymptomatic SARS‐CoV‐2 infections may have occurred before vaccination and remained unrecognized, potentially contributing to the observed antibody and neutralizing antibody responses. Therefore, the magnitude of immune responses attributable solely to booster vaccination could not be fully distinguished from pre‐existing immunity.

Consequently, booster doses of COVID‐19 vaccines provide strong protection against severe disease and mortality despite reduced effectiveness against infection due to emerging variants and waning immunity. In light of evolving variants, the continued use of updated (particularly bivalent or variant‐adapted) vaccines, especially in high‐risk populations, remains essential.

## Author Contributions

**Harika Öykü Dinç:** writing – review and editing, writing – original draft, visualization, supervision, resources, project administration, methodology, investigation, formal analysis, data curation, conceptualization. **Hayriye Kirkoyun. Uysal:** writing – review and editing, validation, resources, methodology, investigation, formal analysis, data curation. **Günay Can:** writing – review and editing, visualization, supervision, software, resources, project administration, methodology, investigation, formal analysis, data curation, conceptualization. **Beyhan Budak:** writing – review and editing, validation, resources, methodology, investigation, formal analysis, data curation. **Ferhat‐Osman Daşdemir:** writing – review and editing, validation, software, resources, methodology, investigation, data curation. **Elif Keskin:** writing – review and editing, validation, resources, methodology, investigation, data curation. **Okan Aydoğan:** writing – review and editing, validation, software, resources, investigation, formal analysis, data curation. **Ilker Inanç Balkan:** writing – review and editing, visualization, supervision, resources, methodology, investigation, data curation, conceptualization. **Rıdvan Karaali:** writing – review and editing, visualization, software, resources, methodology, investigation, data curation. **Suat Sarıbaş:** writing – review and editing, visualization, supervision, resources, methodology, investigation, formal analysis, data curation, conceptualization. **Sevgi Ergin:** writing – review and editing, visualization, supervision, resources, project administration, methodology, investigation, formal analysis, data curation, conceptualization. **Neşe Saltoğlu:** writing – review and editing, visualization, supervision, resources, project administration, methodology, investigation, formal analysis, data curation, conceptualization. **Bekir Kocazeybek:** writing – review and editing, visualization, supervision, resources, project administration, methodology, investigation, funding acquisition, formal analysis, data curation, conceptualization.

## Ethics Statement

This study was approved by Istanbul University‐Cerrahpaşa, Cerrahpaşa Medical Faculty Clinical Research Ethics Committee (Date: Feb 15, 2023, and Decision No: 620145).

## Conflicts of Interest

The authors declare no conflicts of interest.

## Data Availability

The data that support the findings of this study are available on request from the corresponding author. The data are not publicly available due to privacy or ethical restrictions.
